# Small bowel obstruction caused by appendiceal and ileal endometriosis: a case report

**DOI:** 10.1093/jscr/rjac282

**Published:** 2022-06-15

**Authors:** Kazuki Kobayashi, Masato Yamadera, Hiroaki Takeo, Michinori Murayama

**Affiliations:** Department of Surgery, Self-Defence Forces Central Hospital, Setagaya, Japan; Department of Surgery, Self-Defence Forces Central Hospital, Setagaya, Japan; Department of Diagnostic Pathology, Self-Defence Forces Central Hospital, Setagaya, Japan; Department of Surgery, Self-Defence Forces Central Hospital, Setagaya, Japan

**Keywords:** intestinal endometriosis, appendiceal tumour, intestinal obstruction, stenosis

## Abstract

Endometriosis is characterized by the presence of an ectopic endometrial gland. Intestinal endometriosis with small bowel obstruction is uncommon. In this case, a 37-year-old woman with no history of endometriosis presented with a chief complaint of abdominal pain. Computed tomography revealed a distal small bowel obstruction. Surgical intervention was performed because of the emergent abdominal condition and the potential resistance to conservative management. Histopathological examination revealed appendiceal and ileal endometriotic lesions. Preoperative diagnosis was difficult because there were no specific clinical features. Intraoperatively, it is difficult to distinguish intestinal endometriosis and bowel malignancy; thus, oncological resection should be performed.

## INTRODUCTION

Endometriosis is defined as the presence of ectopic endometrial tissue outside the uterine corpus and is estimated to affect 4–17% of menstruating women [1]. Intestinal endometriosis accounts for 3–37% of all endometriosis; and of these, 50–90% affect the rectosigmoid area, 2–16% involve the small bowel and 3–18% affect the appendix [2, 3]. Elective surgical intervention is often performed to treat intestinal endometriosis. Sometimes, urgent medical attention is required in cases with acute abdominal inflammation and bowel obstruction or perforation [1, 2, 4–8]. Endometriosis-related small bowel obstruction is rare. Some reports demonstrated that the rate of intestinal endometriosis causing obstruction is only ~1%, although the true incidence of appendiceal and ileal endometriosis causing bowel obstruction is unknown [9–11]. Herein, we present a case of acute small bowel obstruction secondary to appendiceal and ileal endometriosis requiring emergency surgery.

## CASE REPORT

**Figure 1:**
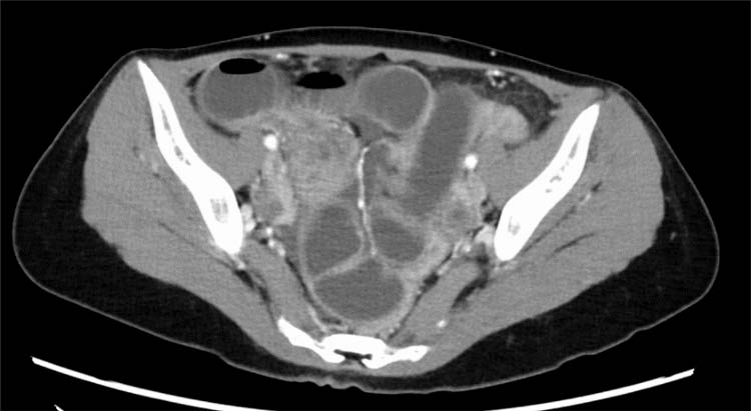
Abdomino-pelvic CT image; dilatation of the small bowel with transition point at the terminal ileum.

**Figure 2:**
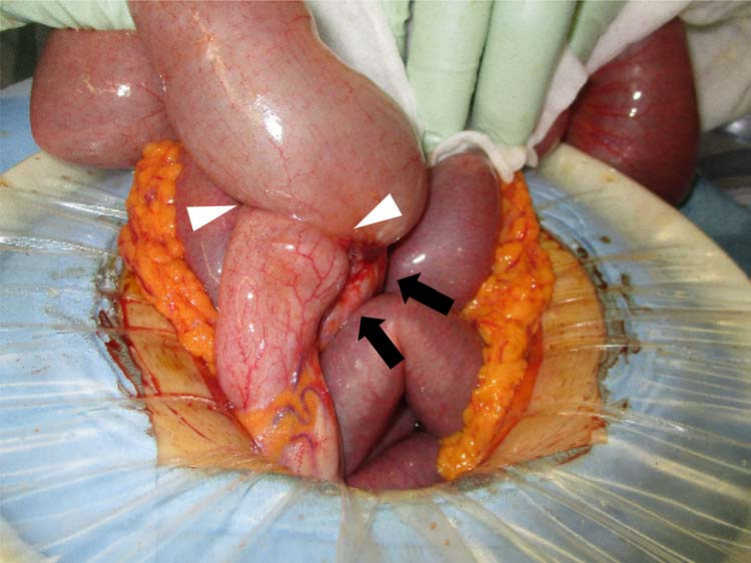
Surgical findings; an appendiceal mass (black arrow) was found and infiltration into the terminal ileum was suspected (white arrowheads).

**Figure 3:**
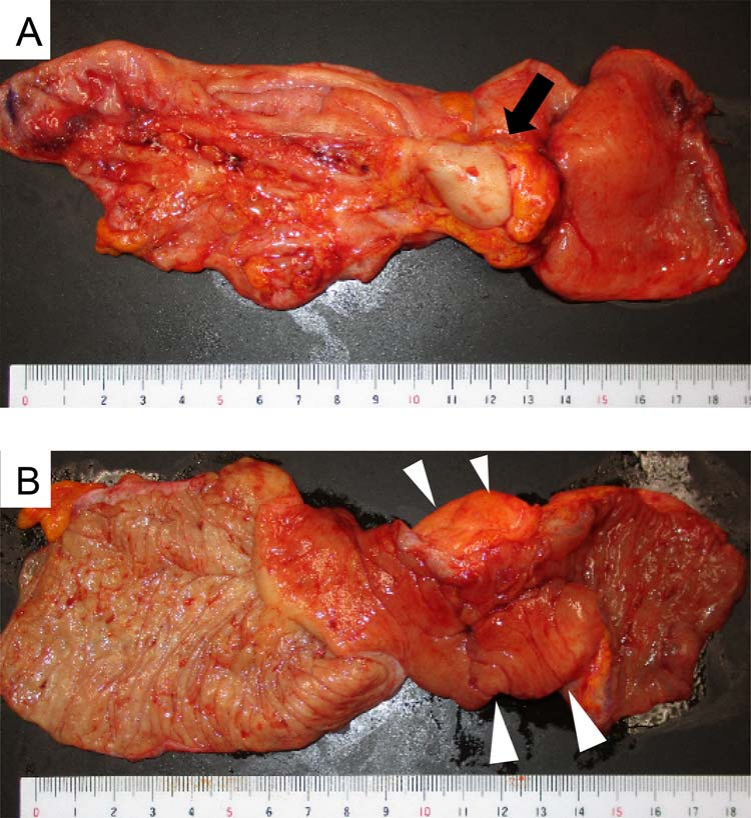
Macroscopic appearance of the ileocecal resected specimen; (**A**) serosal side; swelling of the appendix and mass lesion at the tip of the appendix (black arrow); (**B**) mucosal side; thickening of the terminal ileum wall with no occupied lesion in the mucosa (white arrowheads).

**Figure 4:**
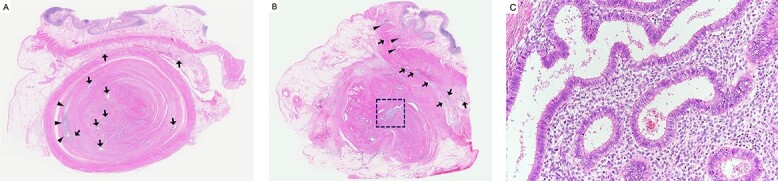
Histopathological findings; (**A**) the appendix shows wall thickening and luminal stenosis by marked fibrosis; the mucosal layer is compressed and remains at the periphery of this cross section (arrowheads); ectopic endometrial glands scatter in the appendiceal wall and submucosa of the ileum (arrow); haematoxylin and eosin (H&E) stain, ×20; (**B**) the lumen and mucosa of the appendix are totally absent in this cross section; a large cluster of ectopic endometrial glands and endometrial stroma is seen in the centre of the appendix (square); ectopic endometrial glands are also seen in the muscularis propria (arrowheads) and subserosa (arrow) of the ileum; H&E stain, ×20; (**C**) a higher magnification image of the square area in (B) shows ectopic endometrial glands surrounded by endometrial stroma; H&E stain, ×200.

A 37-year-old woman presented with an acute onset of diffuse abdominal pain and vomiting unrelated to menstruation. The patient had no history of abdominal surgery, including a caesarean section. Her faecal occult blood test results were positive for the last 3 years, and the annual colonoscopy did not reveal any abnormalities. She had ileus of unknown cause and had undergone conservative medical management at another hospital 1 year prior. Physical examination revealed abdominal distention and peritoneal irritation. Blood examination revealed a slightly elevated circulating white blood cell count of 10 020/μl, but other laboratory data were within the normal range. Computed tomography (CT) revealed ascites and a small bowel obstruction at the level of the distal ileum (Fig. 1). Her abdominal symptoms worsened quickly during the period in which these tests were performed, and she presented with panperitonitis. Therefore, immediate surgical intervention was planned. Intraoperatively, a stricture in the distal ileum, ~5-cm oral from the Bauchin valve, was observed with haemorrhagic ascites. A mass lesion was observed at the tip of the appendix, infiltrating the terminal ileum (Fig. 2). Abnormal findings, such as twitching or pigmentation, were not observed in the serosa at the same site. We performed ileocecal resection with lymph node dissection during complete mesocolic excision, considering a malignant tumour of the intestine. Macroscopically, the resected specimen showed swelling of the appendix, thickening of the intestinal wall and stenosis of the ileum (Fig. 3A and B). Histopathological examination showed fibrous wall thickening and luminal stenosis/obstruction of the appendix by marked fibrosis. Ectopic endometrial glands and stroma were scattered in the thickened appendiceal wall. Fibrous adhesion of the ileum with the appendix was seen, and ectopic endometrial tissues were also scattered in the subserosa and muscularis propria of the ileum at the adhesion site (Fig. 4A–C). These findings were compatible with appendiceal and ileal endometriosis. Sampled lymph nodes did not show findings of endometriosis. The patient was discharged on post-operative day 12 and had an uneventful course without postsurgical hormonal therapy or recurrence of endometriosis.

## DISCUSSION

Herein, we report a rare case of small bowel obstruction with appendiceal and ileal endometriosis. Clinically, the symptoms of intestinal endometriosis are numerous and non-specific [1, 2]. Although visible, pigmented (red or black) serosal lesions, also known as ‘blueberry spots’, are helpful for the intraoperative diagnosis of endometriosis, the present case did not exhibit such lesions [12]. A positive faecal occult blood test result in our case might have implied appendiceal and ileal endometriosis. It is difficult to find abnormalities in the appendix and/or ileum using a colonoscopy alone. If there is a reproducible or cyclical intestinal abnormality of unknown cause in the colonoscopy, additional examinations might be considered.

Intestinal endometriosis is mainly treated surgically because of the difficulty of preoperative diagnosis. Patients with intestinal endometriosis may present with acute abdominal pain and require urgent medical attention [1, 2, 4–8]. Similar to several reports, surgical treatment for malignant tumours was performed in this case because we were not able to diagnose whether the mass lesions in the intestine were benign or malignant [1, 2, 4, 6, 8].

Endometriosis sometimes also involves the lymph nodes; however, according to some reports, the positive lymph nodes that are left untreated might have no clinical influence [5, 13]. The retrieved lymph nodes in our case were all normal, and tumour resection alone may have been the best procedure if intestinal endometriosis was evident.

One of the characteristic findings in our case was that endometrial glands were found in the appendix and ileum of both organs. To the best of our knowledge, only three similar cases have been reported [2, 13, 14]. The cause of the intestinal obstruction in this case was suspected to be an appendiceal mass infiltrating the ileum and creating an intrinsic compression of the terminal ileum. The aetiology of endometriosis remains unknown, but many theories have been proposed to explain this pathology, such as the neurological hypothesis and migration of cells through the lymphatic system or nervous spread [1, 7]. It can also result in caesarean section due to decidua displacement [8]. The most widely accepted theory is ‘retrograde menstruation’, the implantation and growth of endometriosis on the serosal surface of extrauterine organs or secondary to metaplasia in the pelvic peritoneum [15]. The patient in this case had no history of caesarean section, and the aetiology seemed to be compatible with the retrograde menstruation theory. Histological sections showed widely distributed endometrial glands embedded in the lumen and wall of the appendix and in the submucosa and subserosa of the ileum. The ileal endometrial gland was seen mainly in the adhesions between the ileum and the appendix. So, it was considered that the way of endometrial gland development might be involved in adhesions, or it might have occurred simultaneously in the appendix and the distal ileum present nearby.

Appendiceal and ileal endometriosis is rare cause of small bowel obstruction. Preoperative diagnosis is difficult because of the non-specific symptoms and CT findings. Exclusion of bowel malignancy is essential; if doubtful, oncological resection should be performed.

## AUTHORS’ CONTRIBUTIONS

M.Y. and M.M. performed surgeries. K.K. performed post-operative management. K.K., M.Y. and M.M. were involved in drafting the manuscript, revising it critically for important intellectual content and making substantial contributions to clinical data acquisition. H.T. made substantial contributions to the pathological data acquisition and helped draft the manuscript. All authors have read and approved the final manuscript.
